# The need for operational research and capacity-building in support of the Global Technical Strategy for Malaria 2016–2030

**DOI:** 10.1186/s12936-016-1302-x

**Published:** 2016-04-25

**Authors:** Andrew Ramsay, Piero Olliaro, John C. Reeder

**Affiliations:** Special Programme on Research and Training in Tropical Diseases (TDR), a co-sponsored programme of UNICEF/UNDP/World Bank/WHO, based at the World Health Organization, Geneva, Switzerland

The Global Technical Strategy for Malaria 2016–2030 was adopted by the 68th World Health Assembly in May 2015. It emphasizes the importance of scaling up malaria control responses and moving towards elimination [1]. It is anticipated that such scale-up will help countries reduce and, eventually, eliminate the human suffering caused by malaria as well as contribute more broadly to the achievement of the Sustainable Development Goals.

**Fig. 1:**
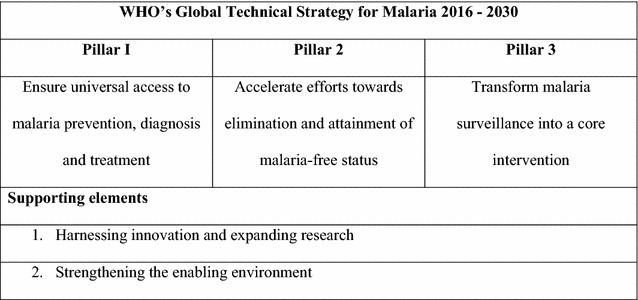
The World Health Organization Global Technical Strategy for Malaria 2016–2030

The Global Technical Strategy consists of three main pillars, underpinned by two supporting elements (Fig. 1). The first of the supporting elements, harnessing innovation and expanding research, is also recognized as critical in the global control and elimination of other diseases, such as tuberculosis [2]. It is recognized that operational and implementation research are needed to ensure that existing interventions are applied effectively and efficiently in different contexts and, as new interventions become available, to ensure that innovation is deployed appropriately and to maximum effect.

In 2011, the malERA Consultative Group on Health Systems and Operational Research published a Research Agenda for Malaria Eradication: Health Systems and Operational Research [3]. The WHO Global Malaria Programme (GMP) has more recently published the report of a multi-partner meeting that discussed operational challenges for malaria elimination, identified priority operational research questions and recommended ways forward [4]. Other organizations have produced similar lists of operational research priorities [5].

There is no shortage of recommendations on the priorities for operational research and most authors consider operational research to be relatively straightforward and, if using routinely-collected data, inexpensive. However, a recent review of the literature on malaria control and elimination between 2008 and 2013 (15,886 articles) revealed that less than 4 % met the definition of operational research. Of these articles only 19 (3.8 %) were related to malaria surveillance [6]. It would seem that operational research, though recognized as critically important to the success of global strategies for malaria control and elimination, is not so commonly undertaken or at least not so commonly published. Indeed, it could be argued that the difference between these two is academic since unpublished research is unlikely to inform widespread scaling up of malaria activities. Why is so little operational research done when much of it would be straightforward and inexpensive and could be done within the context of routine malaria programme activities?

It is a long-standing problem. In 2007, a report of the Global Fund’s Technical Review Panel noted that operational research was often absent or inadequately elaborated in proposals [7]. The report further stated that proposals clearly described bottlenecks to progress and that these provide the basis for operational research questions that seem obvious but were not proposed.

How can operational research be promoted to support the new Global Technical Strategy for Malaria? A good start would be to look at the recent experiences of other disease control programmes. In 2011, the WHO listed its priorities in operational research to improve tuberculosis care and control. One of the five priority areas for operational research was capacity-building for operational research. Key questions included: what are the existing models of operational health research capacity? What is the impact of existing training models in terms of products, outputs and outcomes? How to ensure sustainable operational research capacity at the national level? 4 years later, its Global Action Framework for TB Research described some of the lessons from this exercise and has provided case studies of how operational research capacity has been built in public health programmes in low-income and middle-income countries [2]. One of these case studies was the Structured Operational Research and Training Initiative (SORT IT) a global partnership-based initiative led by the Special Programme for Research and Training in Tropical Diseases (TDR) based at WHO [8]. Started in 2012, SORT IT aims to support countries to: conduct operational research around their own priorities, build adequate and sustainable operational research capacity within public health programmes; and make evidence-informed improvements to programme implementation. More than 400 health workers in 80 countries have been trained through SORT IT to date. Over the course of 1 year, participating health workers learn the skills required to develop operational research questions, protocols, data capture and analysis instruments and to publish their work. Over 90 % of participants publish their research in the peer reviewed literature and many go on to become facilitators in further SORT IT training courses. The training is currently being delivered in English, Russian and Spanish, with the transition into French underway. The model has been recognized as a reliable way to build the much-needed research capacity in public health programmes in LMICs [9, 10].

Having its roots in a training course run by Médecins Sans Frontières and the International Union Against Tuberculosis and Lung Diseases, that supported tuberculosis control, SORT IT is now delivering regional and national capacity-building for operational research in all WHO regions and in number of disease areas [11, 12]. In TDR-supported SORT IT Programmes, the core training course is embedded within broader capacity-building activities in knowledge management (research needs assessments and prioritization, dissemination of research findings and evidence-informed policy making). Efforts are also made to consolidate research capacity that has been built through SORT IT by providing participants with further research training and small research grants.

In 2014, two research studies related to malaria elimination were supported by SORT IT [13, 14]. In 2015–16, in collaboration with the Global Malaria Programme and the WHO African Regional Office (AFRO), a SORT IT programme supported four malaria-eliminating countries in Southern Africa: Botswana, Namibia, South Africa and Swaziland. A Supplement of Malaria Journal is in preparation to feature research outputs of this SORT IT programme. It is hoped that this Supplement will encourage others to adopt approaches like SORT IT, conduct operational research while building research capacity in malaria programmes and make evidence-informed improvements to policy and practice.

On the occasion of the 2016 World Malaria Day the authors would like to highlight both the need for, and the feasibility of, building operational research capacity in malaria programmes in low- and middle-income countries if the ambitions of the Global Technical Strategy for Malaria 2016–2030 are to be achieved.
